# The value of quantitative sensory testing in spine research

**DOI:** 10.1007/s10143-016-0798-4

**Published:** 2016-11-28

**Authors:** Anja Tschugg, Wolfgang N. Löscher, Sara Lener, Sebastian Hartmann, Matthias Wildauer, Sabrina Neururer, Claudius Thomé

**Affiliations:** 1 Department of Neurosurgery, Medical University Innsbruck, Anichstr. 35, A-6020 Innsbruck, Austria; 2 Department of Neurology, Medical University Innsbruck, Innsbruck, Austria; 3 Department of Neuroradiology, Medical University Innsbruck, Innsbruck, Austria; 4 Department of Medical Statistics, Informatics and Health Economics, Medical University Innsbruck, Innsbruck, Austria

**Keywords:** Quantitative sensory testing, Radiculopathy, Lumbar sequestrectomy, Disc herniation, Spine research

## Abstract

The improvement of pain and functionality is the major goal of a surgical intervention. Thus, the purpose of the present prospective study was to evaluate whether subjective sensory deficits in patients with lumbar radiculopathy caused by a lumbar disc herniation are related to clinical status, using several outcome scores and the quantitative sensory testing (QST) pre- and 12 months postoperatively. We applied the QST in 52 patients with a single lumbar disc herniation treated by lumbar sequestrectomy pre- and 12 months postoperatively. Further evaluation included numeric rating scale (NRS) for leg, EuroQoL-5D (EQ-5D), Core Outcome Measure Index (COMI), Oswestry Disability Index (ODI), Beck Depression Inventory (BDI) and PaindDetect questionnaire (PD-Q). Patients were then categorized into two groups based on their subjective recovery of sensory function. The patients’ self-assessment and QST were correlated with each other for the pre- and postoperative visit after 12 months. The two groups showed postoperative differences in mechanical and vibration detection threshold as well as in the postoperative PD-Q (*p* < 0.005). Multidimensional scores did not consistently match the QST parameters in patients with a lumbar disc herniation. Commonly used clinical scores in spine research show low or no correlation with QST. Nevertheless, mechanical thresholds seem to play an important role to detect and follow up a sensory deficit investigated by QST.

## Introduction

Due to the high prevalence of spinal degenerative diseases, the frequency of surgical interventions for degenerative spine pathologies rose dramatically within the last decade [2, 13]. The use of standardized outcome instruments is important to document the treatment effects of surgical interventions and point out potential advantages of new surgical techniques, particularly in the setting of prospective comparative trials. Pre- and postoperative evaluation of pain and pain-related functional impairment represents a major challenge. The most common pain scales and functional scores used in spine research are the numeric rating scale (NRS) and the Oswestry Disability Index (ODI) [7].

In the past decades, quantitative sensory testing (QST) gained popularity as a diagnostic tool to quantify pain and assess sensory function, especially to document a treatment’s effectiveness [9, 10, 16, 19–21]. QST is a psychophysiological test of different sensory modalities that permits a differential assessment of preserved sensation and also of subclinical deficits [4]. Its reproducibility is comparable to that of nerve conduction studies, whereas it has a poor predictive value for identifying the anatomic location of a herniated lumbar disc [16, 21]. However, there is still controversy regarding the current evidence for the association between QST values and the patient’s reported pain and sensory intensity, disability and quality of life [8].

The improvement of pain and functionality is the major goal of a surgical intervention. Moreover, it is important to assess the patient’s postoperative improvement accurately, so that new diagnostic and therapeutic interventions can be applied when needed. Thus, the purpose of this prospective study was to evaluate whether quantitative pain sensation in patients with lumbar radicular pain caused by a lumbar disc herniation is related to clinical status and various outcome scores as well as QST pre- and 12 months postoperatively.

## Material and methods

The study was purely observational, and there were no recommendations for additional diagnostic measures or interventions. Pain management was not delayed or altered by participation in this study. All subjects signed the informed consent form. The study was approved by the Local Ethics Committee in accordance with the ethical principles originating from the Declaration of Helsinki and in compliance with Good Clinical Practice. Patients were considered for inclusion if they had a single-level disc herniation confirmed on MRI, pain and a sensory deficit described as numbness in the corresponding nerve root distribution area of L3 to S1. All patients had an indication for sequestrectomy according to the guidelines of DGNC and DGOOC. No previous back surgery has been performed in any of the patients. None of the included patients presented a history of peripheral nervous system disorders. Neither metabolic nor toxic damage to the peripheral nerves was revealed. All data were recorded the day before surgery and 12 months postoperatively.

### Quantitative sensory testing

The QST was performed pre- and postoperatively by a single investigator. Patients were not distracted during the testing and were given clear and identical instructions. The thermal tests were performed using a Sensory Analyzer TSA-II (Medoc, Israel). Cold and warm detection thresholds were measured first (CDT, WDT) then cold pain and heat pain thresholds (CPT, HPT). The mechanical detection threshold (MDT) was measured with a standardized set of modified von Frey hairs (Somedic, Schweden) that exert forces upon bending between 0.25 and 512 mN. The vibration detection threshold (VDT) was performed with a Rydel-Seifer tuning fork (64 Hz, 8/8 scale). The mechanical pain threshold (MPT) was measured by a custom made pinprick set with forces from 8 to 512 mN. Mechanical pain sensitivity (MPS) was assessed using the same pinprick stimuli to obtain a stimulus response function for pinprick evoked pain. A pressure gauge device (FDK 20, Wagner Instruments, USA) was used to measure the pressure pain threshold (PPT) [14, 15].

### Questionnaire

Standardized spine outcome measures were prospectively evaluated in this study: (1) the numeric rating scale (NRS) for leg pain on a 0–10 rating scale, with higher scores indicating worse pain [7]. (2) The Beck Depression Inventory (BDI) is a multiple choice self-reported inventory for measuring the severity of depression and responsiveness to treatment. A four-point scale indicates the degree of severity. Outcome is as follows: 0–9: minimal depression, 10–18: mild depression, 19–29: moderate depression, 30–63: severe depression [17]. (3) The Core Outcome Measure Index (COMI) is a short, multidimensional outcome instrument, with excellent psychometric properties, that has been recommended for use in monitoring the outcome of spinal surgery from the patient’s perspective. The COMI has one question each on back pain intensity, leg pain intensity, function, symptom-specific well-being, general quality of life, work disability and social disability, scored as a 0–10 index [11, 12]. (4) The generic health status is assessed with the EuroQoL-5Dimension (EQ-5D). The EQ-5D-3L essentially consists of 2 pages—the EQ-5D descriptive system and the EQ visual analogue scale (EQ VAS). The EQ-5D-3L descriptive system comprises the following five dimensions: mobility, self-care, usual activities, pain/discomfort and anxiety/depression. Each dimension has three levels: no problems, some problems and extreme problems. EQ VAS is a vertical 20-cm visual analogue scale with the end points labelled the best imaginable health state at the top and the worst imaginable health state at the bottom having numeric values of 100 and 0, respectively [1]. (5) The Oswestry Disability Index (ODI) is a widely used tool for the assessment of therapeutic effect and is validated and reliable. The ODI consists of ten sections, with six questions in each section. A lower score indicates a higher level of function. An overall score of all ten sections of the ODI will be computed and used as the ODI score. The standardized version of the ODI can be computed by re-scaling the score to the range 0 to 100 [3]. (6) To detect neuropathic pain components and sensory deficits in patients with radicular pain, the PainDETECT questionnaire (PD-Q) was performed. It is a self-report questionnaire with nine items. There are seven weighted sensory descriptor items and two items relating to the spatial (radiating) and temporal characteristics of the individual pain pattern. When using PD-Q for screening purposes, cut-off scores ≤12 (a neuropathic component is unlikely) and ≥19 (a neuropathic component is likely) were found to be the most appropriate [5].

### Statistical analysis

All patients with a complete preliminary examination were considered for inclusion into the cohort. All values were expressed as mean ± SD. The Kolmogorov-Smirnov test was used for testing normal distribution. The unpaired Student’s *t* test, Mann-Whitney *U* test and Fisher’s exact test were used to analyse differences in clinical and demographic characteristics and in clinical outcome variables. Spearman’s rho correlation was performed to assess the relation of patients’self-assessment on pain and QST variables. A *p* value <0.05 was considered statistically significant. All statistical computations were performed with SPSS version 21.0 (IBM Corp. Released 2012. IBM SPSS Statistics for Windows, Version 21.0, NY: IBM Corp.). Figures were designed using GraphPad Prism (version 5.0 for Mac OS X, GraphPad Software, La Jolla California USA, www.graphpad.com).

Statistical evaluations were carried out for the following conditions:

- The preoperative assessment was compared with the postoperative visit after 12 months.
- The patients were categorized into two groups based on their subjective recovery of sensory function (incomplete (persisting hypaesthesia) vs. complete restoration of sensation). Pre- and postoperative assessment was compared as well as any improvement or deterioration during the postoperative course of 12 months.
- The patients’self-assessment and QST parameters were correlated with each other for the pre- and postoperative visit after 12 months.

## Results

Fifty-two patients, 31 men and 21 women, with a single lumbar disc herniation causing lumbar radiculopathy and a sensory deficit, were prospectively included in the trial. The loss to postoperative 6 months follow-up was 1.9 % and to 12 months follow-up was 3.8 %. A recurrent disc herniation occurred in nine (17.3 %) patients, while an accidental durotomy occurred in one patient. These patients were excluded from further statistical analysis as these factors could have influenced the outcome data. Therefore, 38 patients were thus analysed after 12 months follow-up.

The patients’ postoperative course after treatment with lumbar sequestrectomy was assessed using both patients’ self-assessment by different scales and scores and QST parameters. Three stages were observed:

Firstly, patients’ self-assessment and QST parameters demonstrated changes in the patients’ condition during the postoperative follow-up period of 12 months. Lumbar sequestrectomy resulted in a dramatic reduction of leg pain at rest (NRS 6.0 ± 2 vs. 0.8 ± 2) postoperatively (*p* = 0.000). CDT (24.4 °C (±5) vs. 27.8 °C (±2)), MDT (16.5 mN (±18) vs. 9.2 mN (±22)), MPS (1.5 (±2) vs. 2.4 (±2)), VDT (5.5 Hz (±2) vs. 6.6 Hz (±1)) and PPT (6.7 kg (±2) vs. 8.1 kg (±2)) improved from baseline to 12 months follow-up (*p* < 0.005).

**Fig. 1:**
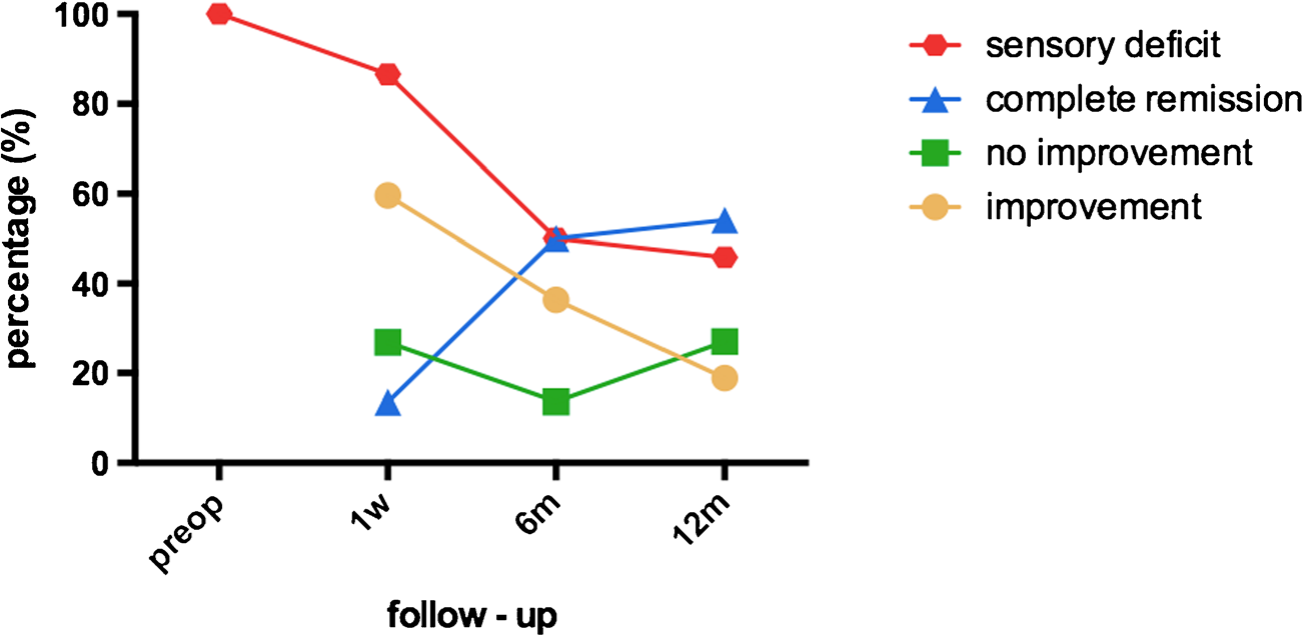
Subjective sensory improvement 1 week, 6 and 12 months postoperatively. Twelve months postoperatively, the patients were categorized into two groups based on their subjective sensory rating. *Prep* preoperative, *w* week, *m* months

**Fig. 2:**
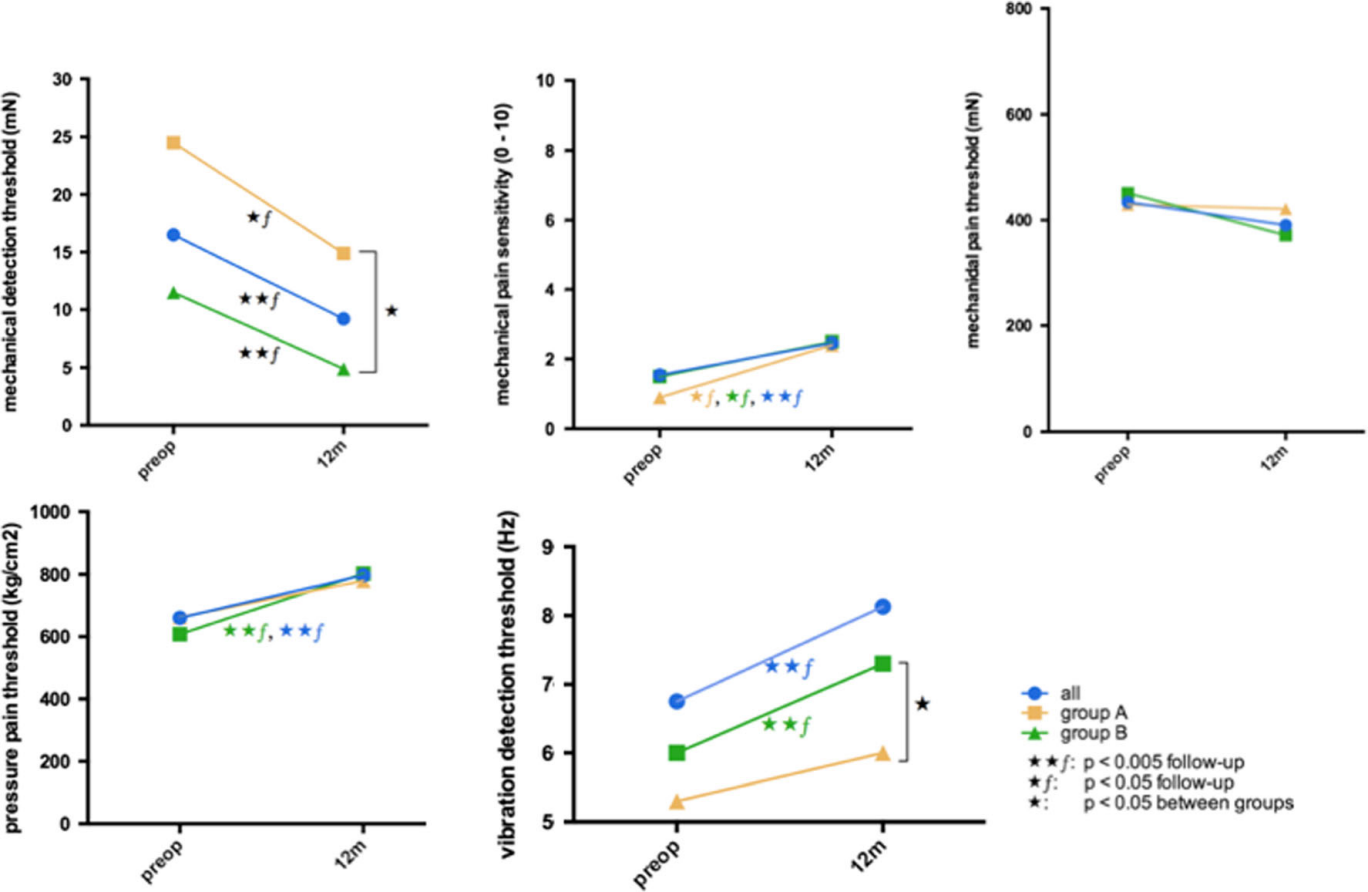
Pre- and postoperative differences in mechanical pain and perception thresholds upon the patients’ subjective rating. *Preop* preoperative, *m* months, *p* significant difference between groups, *p*ƒ = significant difference between follow-up, ⋆*p* < 0.05, ⋆⋆*p* < 0.005 (statistical significance)

**Fig. 3:**
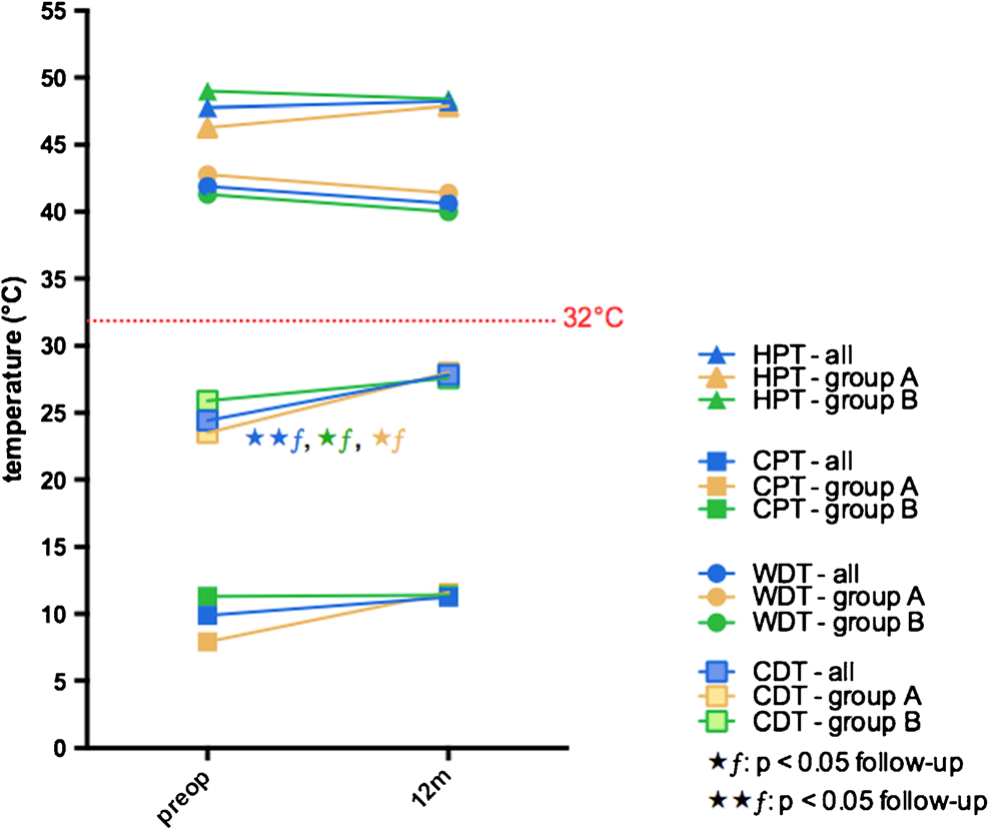
Pre- and postoperative differences in thermal pain and perception thresholds upon the patients’ subjective rating. *CDT* cold detection threshold, *CPT* cold pain threshold, *HPT* heat pain threshold, *WDT* warm detection threshold, *preop* preoperative, *m* months, *p*ƒ significant difference between follow-up, ⋆*p* < 0.05, ⋆⋆*p* < 0.005 (statistical significance)

Secondly, the patients were asked for the subjective sensory improvement 1 week, 6 and 12 months postoperatively. There have been no differences between patients with and without subjective sensory deficits after 1 week and 6 months postoperative (*p* > 0.05). Twelve months postoperatively, the patients were categorized into two groups based on their subjective sensory rating (Fig. 1): (A) with disturbed sensory function (44.7 %) and (B) with complete restoration of sensory function (55.3 %). Allodynia was reported preoperatively in one patient in group A but was not present in any of the patients postoperatively. The results of QST and the patients’ self-assessment are presented in Table 1. The postoperative VDT and MDT revealed to be the only statistically different value in QST testing between groups (VDT: group A: 6.0 Hz (±2) vs. group B: 7.3 Hz (±1); MDT: group A:14.9 mN (±27) vs. group B: 4.9 mN (±17)) (*p* < 0.05), but there were no differences in preoperative QST values between groups A and B (*p* > 0.05). Postoperatively, CDT, MDT, MPS, VDT and PPT improved significantly after surgery in group B, but in group A, an improvement was only measured in CDT, MDT and MPS, respectively (*p* < 0.05) (Figs. 2 and 3). The patients’ self-assessment showed a significant improvement in all scales and scores in both groups (*p* < 0.005). Differences between group A and B were assessed in the PD-Q 12 months postoperatively (group A: 7.8 (±5) vs. group B: 4.0 (±4); *p* < 0.005).

**Table 1:** Pre- and postoperative differences in QST and multidimensional scores

QST parameters	All *n* = 38			Group A (deficit) *n* = 21			Inter-group Pre-op	Inter-group 12 m	Group B (no deficit) *n* = 17		
	Pre-op	12 m	*p* value	Pre-op	12 m	*p* value	*p* value	*p* value	Pre-op	12 m	*p* value
CDT (°C)	24.4 ± 5	27.8 ± 2	**	23.5 ± 7	28.0 ± 2	*	n.s.	n.s.	25.9 ± 3	27.6 ± 2	*
WDT (°C)	41.8 ± 4	40.6 ± 3	n.s.	42.7 ± 3	41.4 ± 3	n.s.	n.s.	n.s.	41.3 ± 4	40.0 ± 3	n.s.
CPT (°C)	9.8 ± 9	11.2 ± 9	n.s.	7.9 ± 9	11.6 ± 10	n.s.	n.s.	n.s.	11.3 ± 9	11.4 ± 9	n.s.
HPT (°C)	47.7 ± 5	48.2 ± 2	n.s.	46.3 ± 9	47.9 ± 2	n.s.	n.s.	n.s.	49.0 ± 1	48.4 ± 1	n.s.
MDT (mN)	16.5 ± 18	9.2 ± 22	**	24.6 ± 23	14.9 ± 27	*	*	n.s.	11.5 ± 12	4.9 ± 17	**
MPT (mN)	433.9 ± 150	390.5 ± 152	n.s.	429.6 ± 161	421.4 ± 126	n.s.	n.s.	n.s.	451.0 ± 137	371.8 ± 171	n.s.
MPS (1–10)	1.5 ± 2	2.4 ± 2	**	0.9 ± 1	2.4 ± 2	*	n.s.	n.s.	1.5 ± 1	2.5 ± 2	*
VDT (Hz)	5.5 ± 2	6.6 ± 1	**	5.3 ± 1	6.0 ± 2	n.s.	n.s.	*	6.0 ± 2	7.3 ± 1	**
PPT (kg/cm2)	660.5 ± 279	797.3 ± 203	**	662.1 ± 285	779.1 ± 219	n.s.	n.s.	n.s.	608.0 ± 221	802.7 ± 194	**
Patients’ self-assessment	All *n* = 38			Group A (deficit) *n* = 21			Inter-group Pre-op	Inter-group 12 m	Group B (no deficit) *n* = 17		
	Pre-op	12 m	*p* value	Pre-op	12 m	*p* value	*p* value	*p* value	Pre-op	12 m	*p* value
NRS leg	6.0 ± 2	0.8 ± 2	*	5.8 ± 3	0.82 ± 2	**	n.s.	n.s.	5.6 ± 2	0.9 ± 2	**
BDI	7.1 ± 5	3.7 ± 5	**	6.6 ± 5	3.4 ± 4	**	n.s.	n.s.	6.2 ± 5	4.0 ± 6	**
ODI	37.3 ± 17	8.2 ± 9	**	39.1 ± 19	9.1 ± 10	**	n.s.	n.s.	35.0 ± 14	7.6 ± 9	**
EQ-5D	0.8 ± 0.7	0.9 ± 0.5	**	0.8 ± 0	0.9 ± 0	**	n.s.	n.s.	0.8 ± 0	0.9 ± 0	**
COMI	6.5 ± 1	1.04 ± 1	**	6.8 ± 1	1.1 ± 1	**	n.s.	n.s.	5.9 ± 1	0.9 ± 1	**
PD-Q	17.8 ± 5.9	5.7 ± 5	**	19.4 ± 6	7.8 ± 5	**	n.s.	*	16.5 ± 5	4 ± 4	**
EQ-5D VAS	5.3 ± 2	8.4 ± 1	**	5.1 ± 2	8.3 ± 1	**	n.s.	n.s.	5.2 ± 2	8.5 ± 2	**

Patients were categorized into two groups based on their subjective sensory rating: (A) with disturbed sensory function and (B) with complete restoration of sensory function. Data is presented as mean ± SD

*CDT* cold detection threshold, *CPT* cold pain threshold, *HPT* heat pain threshold, *MDT* mechanical detection threshold, *MPT* mechanical pain threshold, *MPS* mechanical pain sensitivity, *n.s.* not significant, *PPT* pressure pain threshold, *VDT* vibration detection threshold, *WDT* warm detection threshold, *BDI* Beck Depression Inventory, *COMI* Core Outcome Measure Index, *EQ-5D* Euro-Quality of Life-5Dimension, *NRS* numeric rating scale, *ODI* Oswestry Disability Index, *PD-Q* PainDetect questionnaire

*p < 0.05, **p < 0.005 (statistical significance)

In the third stage, the pre- and postoperative examinations were then correlated with each other (Tables 2 and 3). A correlation could be shown between the BDI, ODI, EQ-5D with the PPT, the ODI and EQ-5D with the MPT, the NRS with the MPS and the PD-Q with the HPT (*p* < 0.05). NRS for leg correlated significantly with ODI, EQ-5D and COMI preoperatively (*p* < 0.05). Postoperatively, EQ-5D, EQ-5D VAS, COMI, PD-Q and ODI showed a significant correlation with NRS for leg (*p* < 0.05).

**Table 2:** Pre- and postoperative correlation between QST and multidimensional scores

Pre-op *n* = 52		CDT	WDT	CPT	HPT	MDT	MPT	MPS	VDT	PPT
NRS leg	*r*	−0.006	0.062	0.227	−0.226	−0.274	−0.245	0.306*	0.123	−0.119
BDI	*r*	−0.103	−0.015	0.045	−0.159	−0.100	−0.134	0.178	0.206	−0.324*
ODI	*r*	0.103	−0.115	0.128	−0.216	−0.096	−0.275*	0.166	0.079	−0.316*
EQ-5D	*r*	0.085	0.022	−0.106	−0.069	−0.042	0.292*	−0.026	−0.118	0.339*
COMI	*r*	0.014	0.128	0.110	−0.028	0.007	−0.218	−0.061	0.027	−0.012
PD-Q	*r*	−0.076	−0.053	0.148	−0.277*	0.008	−0.020	0.007	−0.161	−0.080
EQ-5D VAS	*r*	0.096	−0.105	−0.093	−0.074	−0.227	−0.011	0.221	−0.234	0.122
12 m post-OP *n* = 38		CDT	WDT	CPT	HPT	MDT	MPT	MPS	VDT	PPT
NRS leg	*r*	−0.101	−0.063	0.156	−0.077	−0.055	0.055	−0.010	−0.010	−0.149
BDI	*r*	−0.084	−0.017	0.176	−0.131	0.034	−0.093	−0.140	−0.125	−0.253
ODI	*r*	−0.053	−0.182	−0.151	−0.026	−0.544*	−0.001	−0.357	−0.028	0.174
EQ-5D	*r*	0.046	0.379	0.239	0.254	−0.104	0.215	0.196	0.025	0.292
COMI	*r*	−0.001	−0.175	−0.262	−0.028	0.035	0.011	−0.436*	0.177	0.115
PD-Q	*r*	−0.184	−0.046	−0.156	−0.017	0.066	−0.294	−0.366	0.032	−0.041
EQ-5D VAS	*r*	−0.147	0.361	−0.051	0.197	0.040	0.062	0.157	−0.184	0.286

*CDT* cold detection threshold, *CPT* cold pain threshold, *HPT* heat pain threshold, *MDT* mechanical detection threshold, *MPT* mechanical pain threshold, *MPS* mechanical pain sensitivity, *n.s.* not significant, *PPT* pressure pain threshold, *VDT* vibration detection threshold, *WDT* warm detection threshold, *BDI* Beck Depression Inventory, *COMI* Core Outcome Measure Index, *EQ-5D* Euro-Quality of Life-5Dimension, *NRS* numeric rating scale, *ODI* Oswestry Disability Index, *PD-Q* PainDetect questionnaire, *r* correlation coefficient

*p < 0.05 (statistical significance)

**Table 3:** Pre- and postoperative correlation between multidimensional scores

Pre-OP *n* = 5Pre-OP *n* = 52		NRS leg	BDI	ODI	EQ-5D	COMI	PD-Q	EQ-5D VAS
NRS leg	*r*	1.000	0.140	0.466**	−0.361*	0.363*	0.248	0.048
BDI	*r*	0.140	1.000	0.436**	−0.328*	0.235	0.424**	0.320*
ODI	*r*	0.466**	0.436**	1.000	−0.701**	0.367**	0.333*	0.221
EQ-5D	*r*	−0.361*	−0.328*	−0.701**	1.000	−0.275*	−0.197	0.179
COMI	*r*	0.363*	0.235	0.367**	−0.275*	1.000	0.357**	0.222
PD-Q	*r*	0.248	0.424**	0.333*	−0.197	0.357**	1.000	0.151
EQ-5D VAS	*r*	0.048	−0.030	−0.320*	−0.221	−0.222	−0.151	1.000
Pre-OP *n* = 52		NRS leg	BDI	ODI	EQ-5D	COMI	PD-Q	EQ-5D VAS
NRS leg	*r*	1.000	0.246	0.317*	−0.532**	0.441**	0.385*	−0.464**
BDI	*r*	0.246	1.000	0.485**	−0.518**	0.615**	0.562**	−0.559**
ODI	*r*	0.317*	0.485**	1.000	−0.678**	0.736**	0.522**	−0.601**
EQ-5D	*r*	−0.532**	−0.518**	−0.678**	1.000	−0.719**	−0.543**	0.621**
COMI	*r*	0.441**	0.615**	0.736**	−0.719**	1.000	0.748**	−0.593**
PD-Q	*r*	0.385*	0.562*	0.522**	−0.543**	0.748**	1.000	−0.469**
EQ-5D VAS	*r*	−0.464**	−0.559**	−0.601**	0.621**	−0.593**	−0.469**	1.000

ssion Inventory, *COMI* Core Outcome Measure Index, *EQ-5D* Euro-Quality of Life-5Dimension, *NRS* numeric rating scale, *ODI* Oswestry Disability Index, *PD-Q* PainDetect questionnaire, *r* correlation coefficient

*p < 0.05, **p < 0.005 (statistical significance)

## Discussion

We investigated whether common multidimensional scales and self-reported sensory deficits described as numbness in patients with lumbar radiculopathy are associated with the corresponding QST parameters. Patients demonstrated a postoperative difference in the mechanical perception thresholds according to their postoperative sensory rating. Additionally, postoperative PD-Q showed a significant difference between patients whose sensory deficits recovered and those deficits remained. In contrast, multidimensional scores did not consistently match the QST parameters in patients with a lumbar disc herniation.

It continues to be challenging to assess the patients’ subjective pain and sensation and the extent of pain and of a sensory deficit in an objective and quantitative fashion. Multidimensional scales and scores are used to convey the subjectiveness of pain and sensation into objective quantities in order to measure the success of a surgical intervention or any deterioration after surgery [8]. Thereby, the focus should be placed on patient-orientated measures and that the patient should be the main judge of outcome. Nevertheless, the type of the clinical measurement tested against the QST parameters is essential to answer the same question and avoid misclassification. Questionnaires like the ODI used in this trial preferably asked for the patient’s disability that is an important component of the management of patients with radiculopathy and low back pain [3]. The COMI and EQ-5D assess the multidimensional outcome like for example symptom-specific well-being, general quality of life or social disability in spine surgery [1, 11, 12]. This may be a possible explanation for the lack of a relationship or for the weak correlation between QST values and the questionnaires mentioned above, which by themselves address completely different dimensions of postoperative outcome. QST itself enables the evaluation of a patient’s somatosensory profile accurately and allows to investigate the function of large- and small-fibre sensory modalities [14], especially in follow-up studies [18]. Recovery of sensory function as assessed by QST can be highly relevant for the patient’s satisfaction with surgery.

It is conceivable that numerous factors like pain-related psychological variables account for the variability in pain intensity or disability. Especially depression is an important factor which influences pain and function after surgery [6]. Therefore, patients with depression were excluded from our trial. Nevertheless, a sensory deficit does not seem to influence a patient’s emotional state; particularly it does not give rise to depression.

The PD-Q was originally developed and validated as a screening tool to identify patients with neuropathic pain. QST is a valuable tool to obtain reliable quantitative measures of the presence of positive sensory signs such as allodynia and mechanical or thermal hyperalgesia, as well as negative signs like numbness [2]. Participants with higher PD-Q scores preoperatively were more likely to report a sensory deficit postoperatively. These patients showed significant higher PD-Q scores postoperatively as well. The preoperative PD-Q values in group A showed values over 19, which are highly probable for neuropathic pain [5]. This is explained by one patient that suffered from allodynia preoperatively that completely disappeared postoperatively.

Although there was no correlation between QST values and the PD-Q, it seems that QST values are reflected by responses to verbal descriptors from the PD-Q.

According to the postoperative sensory rating, patients could be categorized in two groups. Upon investigation, there was an improvement in both groups, but still, there was a significant difference in VDT and MDT postoperatively. Thus, our results clearly indicate that mechanical perception thresholds are sensitive and clinically useful parameters, which give quantitative data that may be used in the follow-up of recovery of a sensory deficit [18].

Strengths of our study include the use of the validated, standardized comprehensive DFNS QST protocol, which enables identification of a sensory abnormality within individuals. Furthermore, we are able to present a homogenous study population, while we excluded individuals with major depression or chronic pain/neurological disorders. However, the high loss of follow-up, the small patient cohort and missing control group are the limitations of our study. The fact that there was no correlation between the varying outcome parameters and QST may be especially attributed to the low patient number.

## Conclusion

QST is a structured psychophysical test machinery that is a useful method to differentiate altered sensory and increased pain in patients with lumbar radiculopathy caused by a lumbar disc herniation. Commonly used clinical scores in spine research show low or no correlation with QST parameters. NRS, ODI and EQ-5D are measurements that emerged over the years to assess outcomes because they best reflect the interests of physicians and patients as it concerns current functional status and outcome. Nevertheless, mechanical thresholds like MDT and VDT seem to play an important role to detect and follow a sensory deficit.
